# Wrist function recovers more rapidly after volar locked plating than after external fixation but the outcomes are similar after 1 year: A randomized study of 63 patients with a dorsally displaced fracture of the distal radius

**DOI:** 10.3109/17453674.2011.552781

**Published:** 2011-02-10

**Authors:** Maria K T Wilcke, Hassan Abbaszadegan, Per Y Adolphson

**Affiliations:** Division of Orthopaedics, Karolinska Institutet, Department of Clinical Sciences, Danderyd Hospital, Stockholm, Sweden

## Abstract

**Background and purpose:**

Promising results have been reported after volar locked plating of unstable dorsally displaced distal radius fractures. We investigated whether volar locked plating results in better patient-perceived, objective functional and radiographic outcomes compared to the less invasive external fixation.

**Patients and methods:**

63 patients under 70 years of age, with an unstable extra-articular or non-comminuted intra-articular dorsally displaced distal radius fracture, were randomized to volar locked plating (n = 33) or bridging external fixation. Patient-perceived outcome was assessed with the Disability of the Arm, Shoulder, and Hand (DASH) questionnaire and the Patient-Rated Wrist Evaluation (PRWE) questionnaire.

**Results:**

At 3 and 6 months, the volar plate group had better DASH and PRWE scores but at 12 months the scores were similar. Objective function, measured as grip strength and range of movement, was superior in the volar plate group but the differences diminished and were small at 12 months. Axial length and volar tilt were retained slightly better in the volar plate group.

**Interpretation:**

Volar plate fixation is more advantageous than external fixation, in the early rehabilitation period.

The risk of a poor outcome after a fracture of the distal radius increases with malunion (Grewal and MacDermid 2007) and in highly unstable fractures, operative fixation is required to maintain a satisfactory anatomical position. Closed reduction and bridging external fixation rely on ligamentotaxis to reduce and keep the fracture in alignment. It has been used for unstable distal radius fractures for several decades. External fixation requires 5–6 weeks of immobilization, and some fracture redisplacement often occurs after the fixation device has been removed (Dicpinigaitis et al. 2004, Handoll et al. 2007). In later years, there has been a strong trend towards open reduction and internal fixation with volar locked plating in the management of unstable, dorsally displaced, fractures of the distal radius. Volar locked plating facilitates an anatomical reduction of the fracture, it stabilizes the fracture during the entire healing process, and it allows early wrist mobilization. Good results in terms of patient-rated outcome scores, objective function, and radiographic outcome have been reported both in younger and older patients (Orbay and Fernandez 2002, 2004, Kamano et al. 2005, Chung et al. 2006, Oshiege et al. 2007, Jupiter et al. 2009). Several studies have compared dorsal plating (Grewal et al. 2005, Kateros et al. 2010), fragment-specific systems (Abramo et al. 2009), or a mixture of dorsal and volar plating techniques (Kreder et al. 2005, Leung et al. 2008) with external fixation, but there is no substantial evidence to support the use of internal fixation instead of external fixation (Margaliot et al. 2005). Few studies have compared volar locked plating with external fixation, and there is still insufficient evidence regarding which gives the best outcome (Wright et al. 2005, Egol et al. 2008, Rizzo et al. 2008, Wei et al. 2009).

We have carried out a randomized comparison of open reduction with volar locked plating and closed reduction with bridging external fixation for unstable dorsally displaced extra-articular and non-comminuted intra-articular fractures of the distal radius. Our hypothesis was that volar locked plating would result in better patient-perceived, objective functional, and radiographic outcome after 12 months than external fixation.

## Patients and methods

### Eligibility criteria

The inclusion criteria were: an acute unilateral dorsally displaced fracture of the distal radius (extra-articular AO class A fractures and C1 fractures with only one intra-articular fracture line (Fernandez and Wolfe 2005)), an axial shortening of ≥ 4 mm, or a dorsal angulation of ≥ 20°) in patients aged 20–70 years with no previous fracture of either wrist. Patients were excluded if they had a concurrent fracture of the upper extremities, were medicated with warfarin, were unable to co-operate with follow-up (dementia, substance abuse, psychiatric illness, language problems), had an open fracture or a fracture that was not amenable to both methods, i.e. a distal fragment that was too small (less than 10 mm of intact volar cortex) or too comminuted (AO class C2 and C3 fractures) to allow plate fixation.

### Sample size

The Disabilities of the Arm, Shoulder, and Hand (DASH) score (Hudak et al. 1996) at 12 months was the primary outcome variable used to determine statistical power. The level of significance was set at p < 0.05. Power analysis showed that a 90% power to discover a difference of 10 points (SD 12) in DASH score would require 62 patients. The standard deviation of 12 points was based on data from a group of similar patients in a previous study (Wilcke et al. 2007).

### Ethics

Ethical permission was given by the ethics board of the local university (D.nr. 2005/4:8). Written informed consent was obtained from all patients. The trial was registered at www.ClinicalTrials.gov (NCT00989222).

### Recruitment and randomization

**Figure 1.**
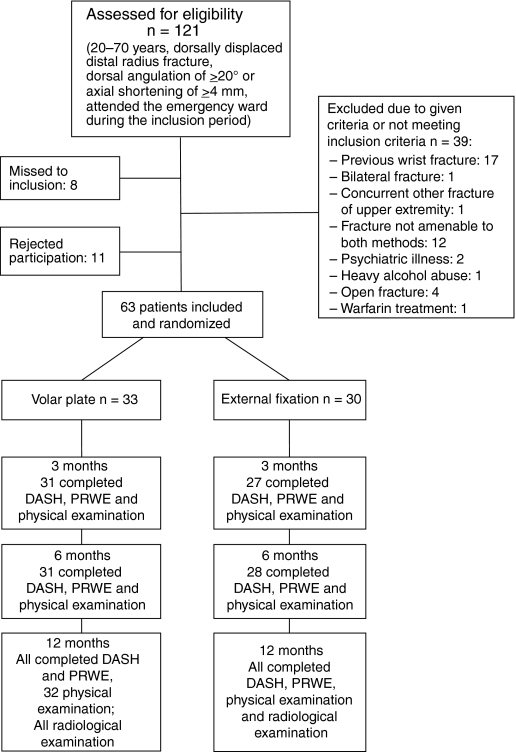
Flow chart of the patients.

During the recruitment period (January 1, 2006 to May 9, 2008), 82 patients met the inclusion criteria (Figure 1). Primary reduction was performed and a temporary plaster cast was applied at the emergency ward. Patients were informed of the study and asked to participate during the visit at the emergency ward or by telephone on the following day. 63 patients were included and randomized by a sealed envelope procedure to either open reduction and internal fixation with a volar locking plate or closed reduction and bridging external fixation. The patients were informed of the randomized method immediately. Randomization was conducted in blocks of 20 with age stratification (over or under 50 years of age).

### Interventions

The patients were planned for day surgery based on the availability of theaters according to normal routines, and the operations were performed after a mean of 4 (1–9) days. The attending trauma surgeon performed the operation; when needed, he/she was assisted by a more experienced colleague. All patients were operated on an out-clinic basis in plexus anesthesia and began finger exercises immediately postoperatively. In plate-fixated patients, a volar flexor carpi radialis approach was used and a volar locked plate with 4 optional distal locked screws was applied (Königsee; Swemac, Sweden). Cancellous bone graft was not used. The patients were given a dorsal below-elbow plaster cast for 10–12 days, after which active wrist mobilization began supervised by a physiotherapist. In patients with external fixation, a Hoffmann device (Stryker, NJ) was applied using 2 pins in the second metacarpal and 2 pins in the radius proximal to the fracture. A closed reduction was performed aided by fluoroscopy, and the fixation device was locked. Supplementary K-wires were not used routinely but at the surgeon's discretion. In 1 case, a radial pin was used. The external fixation was removed after 5 weeks, in the outpatient clinic without anesthesia, and wrist mobilization began supervised by a physiotherapist.

### Outcome assessment

Clinical follow-up was conducted at 10 days, 5 weeks, and 3, 6, and 12 months postoperatively. The validated Swedish versions of the DASH score (Hudak et al. 1996, Atroshi et al. 2000) and the Patient-Rated Wrist Evaluation (PRWE) outcome questionnaire (MacDermid et al. 1998, Wilcke et al. 2009) were used to assess the patient-rated functional result. Both questionnaires yield a score from 0 to 100, where higher scores represent more disability. The questionnaires were completed at baseline, and at 3, 6, and 12 months. Scores are presented with the baseline scores subtracted.

Objective function was assessed with grip strength and range of movement by a physiotherapist at 3, 6, and 12 months. Grip strength was measured with a dynamometer (Grippit; AB Detector, Sweden). The range of movement was measured using a standard goniometer. Both wrists were assessed and the uninjured wrist was used as a control. Grip strength and range of movement are expressed as percentages of those of the uninjured wrist. Grip strength was adjusted by 10% for the non-dominant side (Bechtol 1954). Group assignment could not be blinded to the assessors due to different scars.

**Figure 2.**
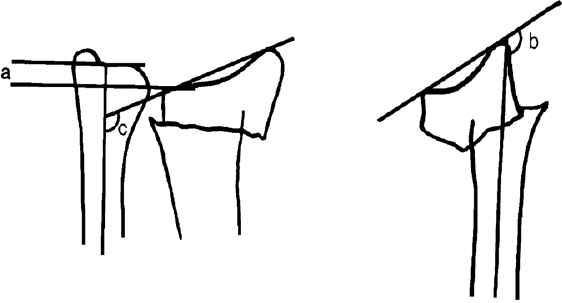
Radiological measurements: axial shortening (a), dorsal angulation (b), and radial angulation (c).

Standard anteroposterior and lateral radiographs were taken directly after surgery, after 5 weeks, and after 12 months. The fractures were classified by one of the authors (MW) according to the AO classification system. Axial shortening, dorsal angulation, and radial angulation were also measured (Figure 2). Radiographs of the uninjured wrist were used as a control. Axial shortening is expressed in mm, and dorsal and radial angulation in degrees. Values are presented as the difference between the injured wrist and the uninjured wrist.

### Statistics

The groups were compared by the Chi-square test for sex, fracture classification, hand dominance, and retirement; Wilcoxon rank sum test for DASH and PRWE scores; and unpaired Student t-test for objective physical and radiographic measurements. The statistical analyses were performed with the statistical package JMP (SAS Institute Inc., NC).

## Results

33 patients were randomized to volar plating and 30 patients to external fixation. The groups were similar in age, sex, and fracture type, and most fractures were extra-articular (Table 1). External fixation was performed by less experienced surgeons to a greater extent than volar plating.

**Table 1.** Clinical data

	Volar plating	External fixation
Mean age (range) in years	55 (20–69)	56 (21–69)
Female, n (%)	25 (76)	23 (77)
AO fracture type: A/C, n (%)	26 (79)/7 (21)	22 (73)/8 (27)
Dominant hand injury, n (%)	15 (45)	14 (47)
Retired, n (%)	9 (28)	9 (30)

### Patient-perceived outcome

The patients in the volar plate group reported statistically significantly better DASH and PRWE scores than the patients in the external fixation group at 3 and 6 months. However, after 12 months, the differences had diminished and were no longer significant (Table 2).

**Table 2.** Patient-perceived results measured by the DASH and PRWE scores

	Volar plating	External fixation	p-value**^a^**
DASH			
3 months	9 (6–12)	27 (20–33)	< 0.001
6 months	6 (3–9)	14 (9–19)	0.008
12 months	7 (4–11)	11 (6–16)	0.1
PRWE			
3 months	14 (8–20)	31 (23–39)	< 0.001
6 months	9 (5–14)	17 (11–22)	0.02
12 months	11 (6–16)	15 (9–21)	0.3

Values are presented as points (95% CI) corrected for baseline values. Higher scores indicate more disability. **^a^** Wilcoxon rank-sum test.

### Objective function

Grip strength and range of movement were superior in the volar plate group but the differences diminished with time (Table 3).

**Table 3.** Objective physical measurements (95% CI) expressed as a percentage of the uninjured side

	Volar plating	External fixation	p-value **^a^**
3 months			
Grip strength	72 (64–80)	46 (37–55)	< 0.001
Extension	84 (78–90)	59 (49–69)	< 0.001
Flexion	81 (77–85)	71 (65–77)	0.009
Ulnar deviation	89 (81–97)	74 (63–86)	0.04
Radial deviation	89 (80–98)	75 (55–95)	0.2
Supination	95 (91–98)	76 (68–85)	< 0.001
Pronation	98 (96–100)	89 (84–94)	< 0.001
6 months			
Grip strength	89 (83–95)	72 (65–78)	< 0.001
Extension	92 (86–97)	77 (68–84)	0.001
Flexion	88 (84–91)	83 (78–88)	0.1
Ulnar deviation	99 (92–106)	91 (79–103)	0.2
Radial deviation	103 (92–115)	89 (70–107)	0.2
Supination	98 (95–100)	88 (84–93)	< 0.001
Pronation	100 (100–100)	95 (92–99)	0.005
12 months			
Grip strength	94 (86–102)	85 (79–91)	0.08
Extension	94 (90–98)	85 (77–93)	0.04
Flexion	89 (86–92)	83 (77–89)	0.08
Ulnar deviation	96 (87–105)	83 (72–93)	0.05
Radial deviation	97 (88–106)	89 (77–100)	0.3
Supination	99 (97–100)	89 (81–98)	0.02
Pronation	99 (98–100)	92 (86–99)	0.04

**^a^** Student's t-test

### Radiographic results

At final follow-up, the patients in the volar plate group had less axial shortening and dorsal angulation of the distal radius than the patients in the external fixation group (Table 4).

**Table 4.** Mean (95% CI) radiological measurements presented as the difference from the uninjured wrist

	Volar plating	External fixation	p-value **^a^**
At injury			
Axial shortening (mm)	4 (3-5)	4 (3-5)	0.9
Dorsal angulation (degrees)	41 (39–44)	43 (40–46)	0.4
Radial angulation (degrees)	11 (8–12)	9 (7–11)	0.5
Post reduction			
Axial shortening (mm)	-2 (-2 to -1)	0 (0–1)	< 0.001
Dorsal angulation (degrees)	6 (5–8)	9 (6–12)	0.1
Radial angulation (degrees)	2 (1–3)	1 (0–2)	0.4
5 weeks postoperatively			
Axial shortening (mm)	0 (-1–0)	1 (1–2)	< 0.001
Dorsal angulation (degrees)	7 (5–9)	11 (7–14)	0.07
Radial angulation (degrees)	2 (1–4)	2 (1–4)	0.9
12 months postoperatively			
Axial shortening (mm)	0 (0–1)	2 (1–3)	< 0.001
Dorsal angulation (degrees)	7 (5–9)	11 (7–15)	0.05
Radial angulation (degrees)	2 (1–4)	3 (1–5)	0.5

**^a^** Student's t-test.

### Complications

In the external fixation group, 1 patient was reoperated with a supplementary volar plate within a week due to an unacceptable fracture position postoperatively. At the 12-month evaluation, a corrective osteotomy was planned in 1 patient, due to a painful malunion. 4 patients suffered from pin tract infections and in 1 of these, pin loosening occurred with malunion as a consequence. 1 patient suffered from a mild complex regional pain syndrome and 1 patient reported a disturbing skin adhesion after a Hoffmann pin. In 4 patients, a light sensory deficit was noted, corresponding to a superficial radial nerve branch.

In the volar plate group, 1 patient was operated on with a median nerve decompression and plate extraction after 6 months due to carpal tunnel syndrome. A transient affection of the median nerve was reported by 3 other patients postoperatively. 1 patient suffered from a rupture of the flexor pollicis longus tendon. This patient started treatment with high-dose corticosteroids during the follow-up period due to a newly discovered cancer. At the 12-month follow-up, 2 patients were planned for plate removal due to irritation from the distal radial edge of the plate and 1 of these patients also had complaints of extensor tenosynovitis superficial to the proximal cortical screws.

## Discussion

We found that volar locked plating was advantageous in the early rehabilitation period, compared to bridging external fixation, but at 1 year the outcomes were similar. Egol et al. (2008) compared volar locked plating and bridging external fixation with supplementary Kirschner wires in 77 patients, and found an improved range of movement early after volar plating but at 1 year the range of movement was similar—as were grip strength and DASH score at any time. These results are in accordance with ours, although we found better grip strength and DASH scores in the plating group at 3 and 6 months. One possible explanation for this difference could be that our plate-fixated patients had a more active early mobilization regime. Another reason for the disparities in DASH scores could be that the variances (standard deviations) were larger in the study by Egol et al. (2008), which might have affected their statistical power negatively. Our external-fixated patients had a somewhat poorer radiographic result than the patients in the study by Egol et al. (2008). This can be explained by the fact that, with 1 exception, our patients did not receive supplementary Kirschner wires since this was not the routine at our department. The use of augmenting pins enhances fragment stability (Dunning et al. 1999) and one could question whether we have compared an insufficient external fixation method with volar plating, and perhaps this would explain why our early findings favored volar plating to a greater extent. However, we do not believe that the small differences in the radiographic results between the groups could account for the differences in grip strength and DASH score in the early rehabilitation period. Rather, we would expect that a worse radiographic outcome would be more likely to affect the result after 1 year. It is interesting however, that even a potentially suboptimal external fixation method gives a similar outcome to that of volar plating after 1 year. By omitting the pins, the risk of radial nerve and skin complications is reduced.

Rozental et al. (2009) randomized 45 patients similar to ours to open reduction and volar plating versus indirect reduction and percutaneous pinning or external fixation. They found that the radiographic outcome was similar between the groups but DASH score, grip strength, and range of movement were superior in the plate-fixated group up to 12 weeks after the injury. The outcomes were similar after 1 year, however. In our material, the plate-fixated patients were also better at 1 year regarding range of movement but the absolute differences were small and probably without any clinical relevance.

Marcheix et al. (2010) randomized 103 patients over the age of 50 years with unstable extra- and intra-articular fractures (including comminuted C2 and C3 fractures) to volar locked plating or “mixed pinning”. At 3 and 6 months, the plated patients had better objective functional results and reported better DASH scores, in accordance with our findings. The 1-year results were not reported.

Wei et al. (2009) compared external fixation (22 patients) with locked radial (12 patients) or volar plating (12 patients) and found that volar-plated patients had better DASH scores in the first 3 months. At 6 and 12 months, however, the DASH scores were similar between the groups.

Our interpretation is that the more rapid recovery of patient-perceived and objective wrist function in patients treated with volar locked plating, compared to external fixation, found by us and by the other authors mentioned above, is due to the earlier active wrist mobilization postoperatively that is allowed by the volar locked plating technique.

A limitation of our study is that it may have been underpowered. It can be argued that we might have found a statistically significant difference in DASH and PRWE scores at 12 months if the patient groups had been larger. However, a retrospective power analysis showed that 70–110 patients in each group would be required to make the differences significant. Considering the small absolute differences at 12 months and the findings of other investigators, as discussed above, we believe that our results are valid.

We used a volar plate with a simple construction, which limited the type of fractures that could be included to extra-articular and non-comminuted AO type C1 fractures. Considering the later risk of osteoarthritis, open reduction and volar plating may also be advantageous in the long term for more complex intra-articular fractures compared to external fixation, since the method allows specific fragment reduction. However, the clinical relevance of radiographic degenerative changes in the wrist is being debated (Downing and Karantana 2008) and there is still little evidence in the literature that volar plating is better than external fixation for intra-articular fractures (Wright et al. 2005, Rizzo et al. 2008).

As the patient-rated function appears to be similar between volar plating and external fixation at 1 year, it is important to consider the complications of the 2 methods. Our study was performed when the volar locked plating method was new to our department; therefore, the good results and relatively few reported complications in the plate group—despite the simple and rather bulky design of the plate—in combination with the learning curve for our surgeons, imply that volar plating is a forgiving method. However, high complication rates have been reported by other authors (Rozental and Blazar 2006, Casaletto et al. 2009, Knight et al. 2010) after volar plating, with fracture collapse and rupture of the extensor pollicis longus and flexor pollicis longus tendons. We did not observe any fracture collapse, perhaps because we did not include the most osteoporotic elderly patients or comminuted intra-articular fractures. 1 patient in our material suffered from a flexor pollicis tendon rupture. If the follow-up period had been longer, we might have seen further cases since this complication can present several years after the fracture (Casaletto et al. 2009).

In the external fixation group, the serious complications were 2 cases of fracture collapse with malunion. Concerning the complications, we do not believe that our study has demonstrated that either treatment has a clear advantage. Randomization was not performed in the operating theater, with the possibility of surgeon bias. To a large extent, external fixation was performed by less experienced surgeons than was volar plating. This may have contributed to the slightly poorer radiographic result in this group and the 2 cases of fracture collapse.

The large number of volar plates that have been implanted in recent years may result in unexpected late complications in the future. What remains to be evaluated is the long-term risk of rupture of the flexor pollicis longus tendon after volar plating. It is a serious complication that requires tendon transfer with a long rehabilitation period. Furthermore, one can speculate that remaining volar plates might potentially lead to more complicated fracture patterns or other problems after a subsequent wrist trauma, especially since patients will age and their osteoporosis will increase. The advantage of external fixation is that complications can be expected to occur within the first year after the injury. Thus, a relevant question is whether the earlier recovery of wrist function after volar plating might be gained at the expense of serious late complications.

The clinical implication of our results is that for unstable extra-articular and simple intra-articular fractures of the distal radius in a patient under the age of 70 years, volar plating should be considered when rapid recovery of wrist function is important. However, overlooking the slower return of wrist function, external fixation is still an effective, inexpensive, and less invasive method.
